# The influence of N-acetyl-L-cysteine on oxidative stress and nitric oxide synthesis in stimulated macrophages treated with a mustard gas analogue

**DOI:** 10.1186/1471-2121-9-33

**Published:** 2008-06-20

**Authors:** Victor Paromov, Min Qui, Hongsong Yang, Milton Smith, William L Stone

**Affiliations:** 1 Department of Pediatrics, College of Medicine, East Tennessee State University, Johnson City, TN 37614, USA; 2 AMAOX, Ltd., #208, 6300 N. Wickham Road, Melbourne, FL 32944, USA

## Abstract

**Background:**

Sulphur mustard gas, 2, 2'-dichlorodiethyl sulphide (HD), is a chemical warfare agent. Both mustard gas and its monofunctional analogue, 2-chloroethyl ethyl sulphide (CEES), are alkylating agents that react with and diminish cellular thiols and are highly toxic. Previously, we reported that lipopolysaccharide (LPS) significantly enhances the cytotoxicity of CEES in murine RAW 264.7 macrophages and that CEES transiently inhibits nitric oxide (NO) production via suppression of inducible NO synthase (iNOS) protein expression. NO generation is an important factor in wound healing. In this paper, we explored the hypotheses that LPS increases CEES toxicity by increasing oxidative stress and that treatment with N-acetyl-L-cysteine (NAC) would block LPS induced oxidative stress and protect against loss of NO production. NAC stimulates glutathione (GSH) synthesis and also acts directly as a free radical scavenger. The potential therapeutic use of the antibiotic, polymyxin B, was also evaluated since it binds to LPS and could thereby block the enhancement of CEES toxicity by LPS and also inhibit the secondary infections characteristic of HD/CEES wounds.

**Results:**

We found that 10 mM NAC, when administered simultaneously or prior to treatment with 500 μM CEES, increased the viability of LPS stimulated macrophages. Surprisingly, NAC failed to protect LPS stimulated macrophages from CEES induced loss of NO production. Macrophages treated with both LPS and CEES show increased oxidative stress parameters (cellular thiol depletion and increased protein carbonyl levels). NAC effectively protected RAW 264.7 cells simultaneously treated with CEES and LPS from GSH loss and oxidative stress. Polymyxin B was found to partially block nitric oxide production and diminish CEES toxicity in LPS-treated macrophages.

**Conclusion:**

The present study shows that oxidative stress is an important mechanism contributing to CEES toxicity in LPS stimulated macrophages and supports the notion that antioxidants could play a therapeutic role in preventing mustard gas toxicity. Although NAC reduced oxidative stress in LPS stimulated macrophages treated with CEES, it did not reverse CEES-induced loss of NO production. NAC and polymyxin B were found to help prevent CEES toxicity in LPS-treated macrophages.

## Background

Mustard gas (HD) is a chemical weapon that can easily and inexpensively be produced and used against military or civilian populations with both acute and devastating long-term effects [1-3]. It produces rapid damage to eyes, skin and pulmonary tissues as well as subsequent damage to many internal organ systems [1,4]. Despite its long history of use, starting in World War I, the molecular mechanisms for HD toxicity are not fully understood and there is continuing research on the design of optimal countermeasures. Mustard gas acts as an alkylating agent covalently modifying DNA, proteins and other macromolecules. There is increasing evidence that HD or CEES toxicity is due, in part, to an enhanced production of inflammatory cytokines [5-9], increased oxidative stress [10] and the generation of damaging reactive oxygen species (ROS) [8,9,11]. HD and CEES have been shown to shift the intracellular redox milieu toward a more oxidized state by reacting with and depleting the intracellular antioxidant GSH with a subsequent loss of protection against ROS and an activation of inflammatory responses [12-14].

In a previous publication, we showed that the cytotoxicity of CEES towards murine RAW 264.7 macrophages was markedly enhanced by the presence of low levels of LPS (25 ng/ml), or pro-inflammatory cytokines, i.e., 50 ng/ml IL-1β or 50 ng/ml TNF-α [15]. LPS is part of the cell wall of gram negative bacteria: it is ubiquitous and is found in serum, tap water and dust. Both civilian and military personnel would always have some degree of exposure to environmental LPS. HD induced skin lesions often have secondary infections which could markedly increase LPS levels. In macrophages, stimulation by LPS, as well as by pro-inflammatory cytokines, leads to the activation and nuclear translocation of transcription factor NF-κB (nuclear factor-kappa B). One of the major consequences of such activation in macrophages is an induction of iNOS expression with subsequent elevation of intracellular NO [16,17]. In addition to NF-κB activation, the binding of transcription factor STAT-1 (signal transducer and activator of transcription-1) to the inducible nitric oxide synthase (iNOS) promoter is required for optimal induction of the iNOS gene by LPS [17].

In a recent publication, we found that CEES transiently inhibits nitric oxide (NO) production by suppressing iNOS protein expression in LPS stimulated macrophages [18]. NO production is an important factor in promoting wound healing [19,20] and iNOS deficiency impairs wound healing in animal models [21]. RAW 264.7 macrophages have undetectable levels of iNOS or NO production in the absence of LPS and in the presence of LPS they show a marked induction of iNOS and NO production [18].

In the present study, we tested the hypothesis that the synergistic cytotoxic effect of CEES with LPS is due to increased oxidative stress with a subsequent depletion of intracellular GSH levels and an increase in protein carbonyls. In some cell types, GSH has also been found to regulate NO generation with decreased GSH levels associated with decreased NO production [22-24]. Vos et al. [25] found that GSH depletion in hepatocytes prevented iNOS induction by cytokines but this effect could be reversed by the addition of NAC. We, therefore, hypothesized that the addition of NAC to stimulated macrophages would reverse the loss of NO production caused by CEES. We also reasoned that polymyxin B, by binding to LPS, would diminish CEES toxicity in LPS treated macrophages.

## Results

### The influence of NAC on cell viability and NO production in CEES/LPS treated macrophages

**Figure 1:**
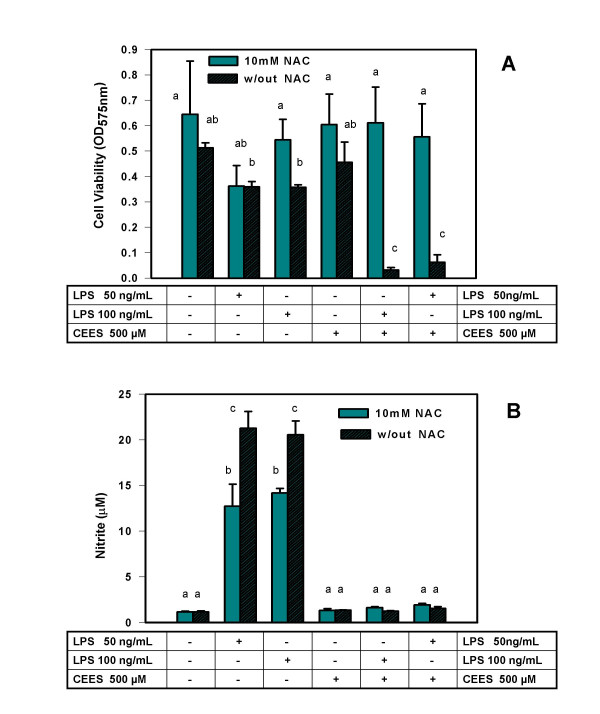
**NAC effect on viability and NO production in CEES/LPS treated RAW 264.7 cells (simultaneous NAC/CEES/LPS application)**. *Panel A: *Macrophages incubated with 50 or 100 ng/ml of LPS or/and 500 μM CEES were simultaneously treated with or without 10 mM NAC (as indicated) for 24 hours. Cell viability was measured using the MTT assay (see Materials and Methods) and expressed as OD at 575 nm. *Panel B: *Macrophages were incubated as described above and NO production measured as the concentration of nitrite in the culture media as described in Materials and Methods. Mean values not sharing a common letter are significantly different (p < 0.05).

Figure 1a shows the effect of NAC treatment on RAW 264.7 macrophages treated with LPS and/or 500 μM CEES for 24 h. In this experiment, NAC was added simultaneously with LPS and CEES. In the absence of NAC, LPS, at either 50 ng/ml or 100 ng/ml level, markedly decreased cell viability in CEES treated cells compared to cells treated with LPS or CEES alone. This is similar to our previous observations in which cell viability was measured by both the MTT assay and the propidium iodide exclusion assay; the assays were well correlated with each other [15]. The addition of 10 mM NAC increased the viability of macrophages treated with both CEES and LPS (50 ng/ml or 100 ng/ml) to the same level observed for control cells (treated with vehicle alone). It is likely that the differences in the viability of cells treated with NAC and different levels of LPS represent experimental variability since these differences are marginal.

Figure 1b shows NO release, measured as the nitrite levels in the cell culture medium, for the identical cells/treatments used in Figure 1a. As expected, LPS treatment alone resulted in a marked increase of NO generation, and LPS-stimulated macrophages treated with CEES showed a marked reduction in NO production. Surprisingly, NAC treatment did not prevent the decrease in NO production caused by CEES. In cells treated with LPS alone, NAC treatment actually resulted in a decreased production of NO (up to 40% reduction).

**Figure 2:**
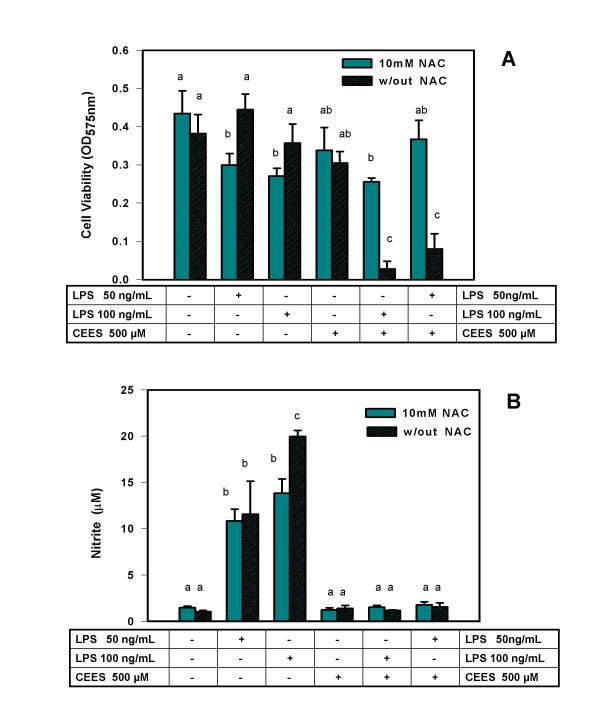
**NAC effect on viability and NO production in CEES/LPS incubated RAW 264.7 cells (NAC pre-treatment)**. *Panel A: *Macrophages were pre-treated with or without 10 mM NAC for 5 hours and then incubated with 50 or 100 ng/ml of LPS or/and 500 μM CEES (as indicated) for 24 hours. Cell viability was measured using the MTT assay (see Materials and Methods) and expressed as OD at 575 nm. *Panel B: *Macrophages were incubated as described above and NO production measured as concentration of nitrite in the culture media as described in Materials and Methods. Mean values not sharing a common letter are significantly different (p < 0.05).

**Figure 3:**
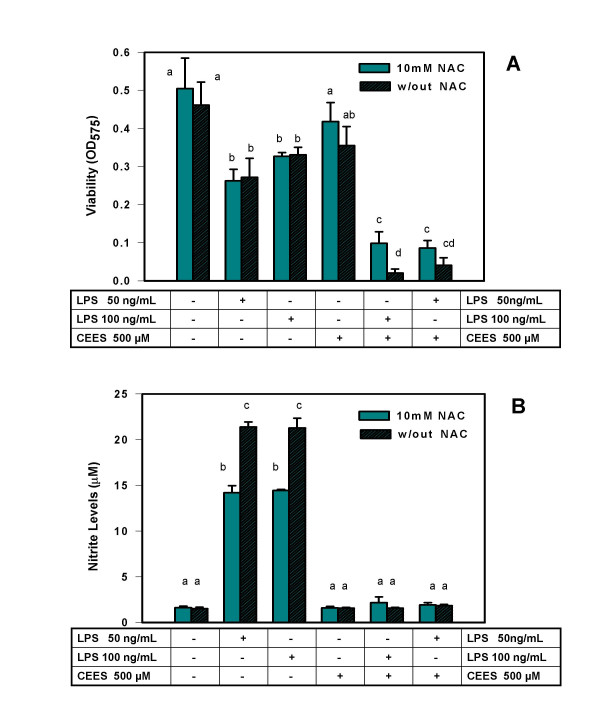
**NAC effect on viability and NO production in CEES/LPS treated RAW 264.7 cells (NAC post-treatment)**. *Panel A: *Macrophages were incubated with 50 or 100 ng/ml of LPS or/and 500 μM CEES (as indicated) for 24 hours and 10 mM NAC was added to the cell culture medium 5 hours after the CEES/LPS application. Cell viability was measured using the MTT assay (see Materials and Methods) and expressed as OD at 575 nm. *Panel B: *Macrophages were incubated as described above and NO production measured as concentration of nitrite in the culture media as described in Materials and Methods. Mean values not sharing a common letter are significantly different (p < 0.05).

In order to further evaluate NAC as a potential protective agent for CEES toxicity in stimulated macrophages, we did two additional experiments in which NAC was added to macrophages 5 h prior to CEES application or 5 h after CEES application. These additional experiments provide a measure of the potential time frame during which NAC could be therapeutically useful. Similar to the previous experiment, LPS and CEES were added simultaneously (as indicated). As shown in Figure 2a, NAC had a substantial protective effect on cell viability when added 5 h before CEES/LPS; however NAC did not protect against loss of NO production in CEES/LPS-treated cells (Figure 2b). When added 5 h after CEES treatment (Figure 3a), NAC was much less effective in protecting the macrophages but still resulted in at least a doubling of the cell viability compared to the cells not treated with NAC. As shown in Figure 3b, NAC added 5 h after CEES/LPS, also failed to restore NO production.

We believe that the difference in viability of cells stimulated with LPS in the absence or in the presence of NAC (Figure 2A) could represent experimental variation since relatively small differences are being compared. In the contrast, the protective effect of NAC on CEES+LPS treated macrophages is robust and over seven fold. This point is further reinforced by the data shown in Figure 3A, where viability of cells stimulated with LPS in the absence or in the presence of NAC was not significantly different.

### The influence of NAC on oxidative stress and NO production, intracellular GSH and thiols in CEES/LPS treated macrophages by fluorescence microscopy

The influence of NAC on macrophages treated with CEES/LPS was also examined by fluorescent microscopy using three fluorescent probes: a) carboxy-dichlorofluorescin diacetate (carDCFH-DA), a sensor for combined ROS and reactive nitrogen oxide species (RNOS) generation [26-28]; b) 7-amino-4-chloromethylcoumarin (CMAC), an indicator of intracellular GSH [29], and; c) 5-chloromethylfluorescein diacetate (CMF-DA), a probe for total non-protein cellular thiol levels that lacks specificity for GSH [29,30].

**Figure 4:**
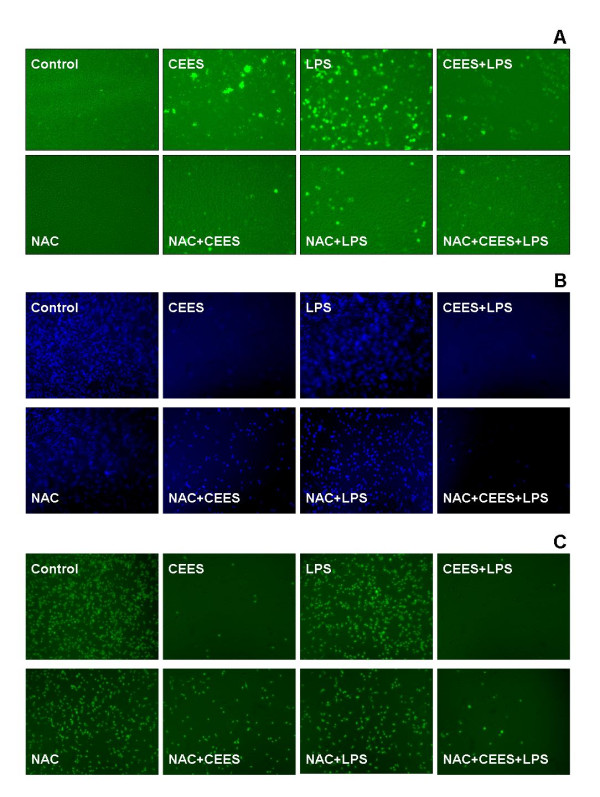
**Fluorescent microscopy probes for oxidative stress, GSH and total thiols in RAW 264.7 cells**. *Panel A*: Combined generation of ROS and RNOS were monitored using 20 μM carDCFH-DA; *Panel B*: Intracellular GSH levels were examined using 20 μM CMAC; *Panel C*: Levels of non-protein cellular thiols were monitored using 20 μM CMF-DA under a fluorescent microscope. Macrophages were treated with CEES (500 μM) and/or LPS (50 ng/mL) and incubated in the absence of NAC (top row in each panel) or in the presence of 10 mM NAC for 12 h.

Figure 4a shows the results using the lipid soluble carDCFH-DA probe. This probe enters cells and is trapped after being converted to a nonfluorescent polar derivative by cellular esterases. CarDCFH can then be oxidized by either ROS [26,28] or reactive nitrogen oxide species (RNOS) [26,27] to the fluorescent product carboxydichlorofluorescein (car-DCF) and thereby provide a qualitative index of oxidation stress. As expected, treatment with LPS alone (50 ng/ml for 12 h) induced a marked generation of ROS plus RNOS in macrophages. We and others have shown that car-DCF fluorescence in activated macrophages is almost entirely from NO generation rather than ROS generation [18,27]. Figure 4a also shows that a 12 h treatment with CEES alone (500 μM) or simultaneous treatment with 500 μM CEES and 50 ng/ml LPS (CEES+LPS) induces a higher level of car-DCF fluorescence than observed in control cells treated with vehicle alone. We previously reported that CEES markedly reduces NO generation in LPS stimulated cells by reducing the expression of inducible iNOS [18]. The car-DCF fluorescence observed in CEES treated cells or CEES+LPS cells is likely, therefore, to be due to an enhanced generation of ROS alone with a minimal contribution from RNOS.

Simultaneous treatment with 10 mM NAC reduced the car-DCF fluorescence observed in LPS stimulated cells, as well as in CEES or CEES+LPS treated RAW 264.7 macrophages (Figure 4a, compare top row to bottom row). These data qualitatively suggest that CEES and CEES+LPS treatments induce oxidative stress in RAW 264.7 macrophages that can be diminished by NAC treatment.

As a next step we examined intracellular levels of GSH using the CMAC probe (Figure 4b, top row) and levels of total intracellular thiols using the CMF-DA probe (Figure 4c, top row). Both the CMAC and CMF probes revealed similar qualitative patterns: CEES or CEES+LPS treatment for 12 h caused cellular GSH and thiol depletion but treatment with LPS alone did not. These data reinforce the notion that treatment with either CEES alone or treatment with CEES+LPS induces sufficient oxidative stress to reduce intracellular GSH and thiol levels. LPS alone, however, did not induce GSH or thiol depletion. NAC application was found to inhibit the loss of GSH and thiol levels caused by CEES or CEES+LPS treatment (see Figures 4b and 4c, bottom rows).

The microscopic data (Figure 4) show merged visible/fluorescent images, thus allowing cell counting and the monitoring of cell morphology changes. The counts of live (morphologically unchanged) cells under conditions described above (Figure 4) confirmed the major observations from the MTT-derived data (Figure 1a): (1) NAC treatment enhance (3-fold) the viability of CEES+LPS treated macrophages; (2) CEES+LPS is more toxic than CEES alone. The cells count (as percentage of untreated control cells ± SEM) were 57% ± 7, 76% ± 10, 18% ± 4, 84% ± 8, 75% ± 9, 67% ± 6, 54% ± 10, respectively for macrophages treated with CEES (500 μM), LPS (50 ng/ml), CEES+LPS, NAC (10 mM), NAC+CEES, NAC+LPS and NAC+CEES+LPS.

### Quantitative effects of CEES on GSH status and protein carbonyl levels in LPS-stimulated RAW 264.7 macrophages

**Figure 5:**
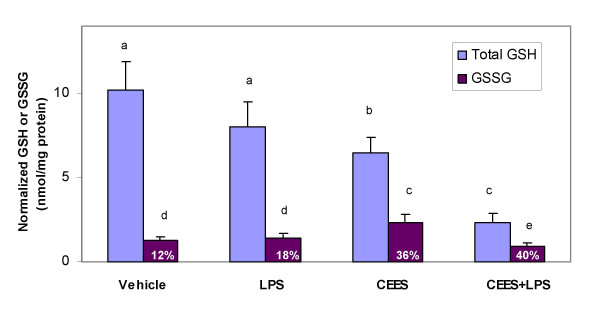
**Glutathione status in RAW 264.7 cells incubated with CEES/LPS**. Macrophages were incubated with 50 ng/ml LPS or/and 500 μM CEES for 12 h. Total GSH (GSH+GSSG) and GSSG levels were measured using a GSH assay kit (see Materials and Methods section) in cell lysates and normalized to total protein. Numbers show the percentage of GSSG in total GSH. Mean values not sharing a common letter are significantly different (p < 0.05).

Since the fluorescence microscopy data presented above are primarily qualitative, we wanted to confirm our results by a more quantitative approach. We, therefore, determined the effect of 500 μM CEES on the GSH/GSSG status of RAW 264.7 macrophage treated or untreated with 50 ng/ml LPS for 12 h. Total GSH (GSH+GSSG) and GSSG concentrations were measured in cell lysates using a quantitative GSH assay kit and the values normalized to total protein content of the lysate (see Materials and Methods). Figure 5 shows that both total GSH and GSSG levels in macrophages treated with either vehicle alone or LPS were not significantly different, i.e., similar to our fluorescent microscopy data. However, cells treated with CEES alone showed a depletion in total GSH as well as an increase in GSSG levels; cells treated with both CEES and LPS were further depleted in total GSH and the percentage of GSSG in these cells was the highest (40%). These results show that LPS alone does not induce a significant oxidative stress, CEES alone induces a moderate oxidative stress but the combination of both CEES and LPS induces the highest observed level of oxidative stress.

In addition, we measured the protein carbonyl levels in control cells, cells treated with CEES (500 μM) alone or cells simultaneously treated with both LPS (50 ng/ml) and CEES (500 μM) for 12 h. Protein carbonyls are stable protein oxidation products. The combination of CEES and LPS produced about 1.5 fold increase in protein carbonyl levels, however cells treated with CEES alone were not significantly different from control cells treated with vehicle alone (data not shown). Cells treated with LPS alone were not assayed in this experiment since both our qualitative (Figure 4b and 4c) and quantitative data (Figure 5) showed no evidence of oxidative stress with this treatment.

### The inability of NAC to reverse NO loss in CEES/LPS treated cells is not GSH dependent

**Figure 6:**
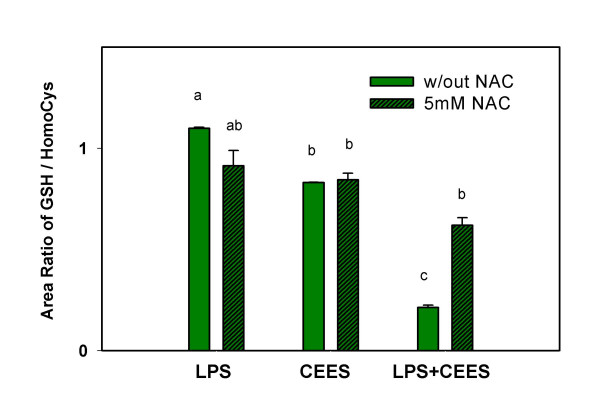
**NAC restores intracellular GSH in RAW 264.7 cells incubated with CEES/LPS**. Macrophages incubated with 50 ng/ml LPS or/and 500 μM CEES were simultaneously treated with or without 5 mM NAC (as indicated). Cell lysates were measured for reduced GSH after 4 hour incubation using the HPLC technique described in Materials and Methods. The GSH levels were normalized to an internal homocysteine standard. Mean values not sharing a common letter are significantly different (p < 0.05).

The data in Figure 1b show that NAC has almost no ability to restore NO production in LPS-stimulated macrophages treated with CEES. An inability of NAC to prevent the depletion of GSH in LPS-stimulated cells treated with CEES could possibly explain these results. In order to explore this possibility, we examined the ability of 5 mM NAC to prevent GSH depletion in LPS (50 ng/ml) stimulated and CEES treated (500 μM for 4 h) RAW 264.7 cells. Figure 6 shows that CEES treatment alone decreased intracellular GSH by only about 10% compared to LPS stimulated cells in the absence NAC. As expected, the decrease in GSH levels was quite large in cells treated with both CEES+LPS (in the absence of NAC) but treatment with 5 mM NAC was effective in preventing this loss. The data shown in Figure 6 were obtained by HPLC analyses of the cell lysates but similar results were obtained by using a fluorometric assay for GSH [31] (data not shown). Despite the fact that NAC can increase the GSH level by three fold in CEES+LPS treated cells it does almost nothing to increase NO production (Figure 1a). These data suggest that the loss of NO production in CEES treated stimulated macrophages is not GSH dependent as has been observed in some other cell lines [22-25].

### Polymyxin B diminishes CEES toxicity in LPS-treated macrophages and partially blocks LPS induced NO production

**Figure 7:**
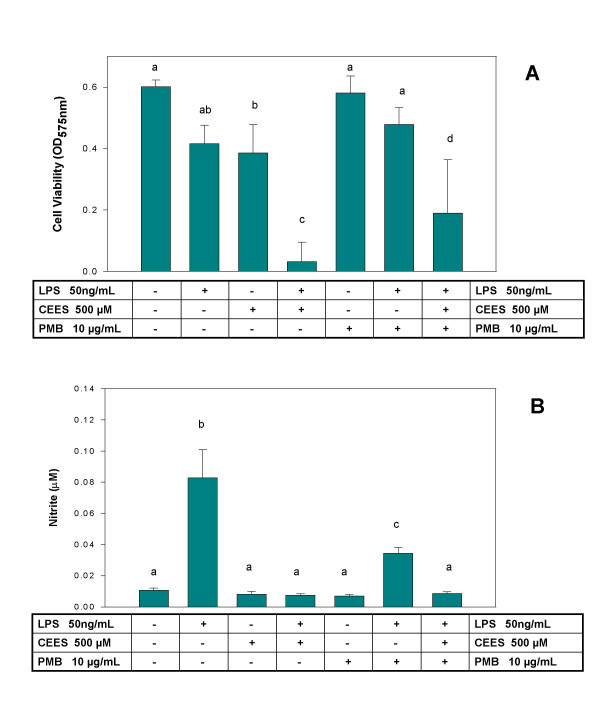
**Polmyxin B partially protects LPS stimulated RAW 264.7 cells from CEES toxicity and blocks NO production**. *Panel A*: Macrophages incubated with 50 ng/ml LPS or/and 500 μM CEES were simultaneously treated with or without 10 μg/mL polymyxin B (as indicated) for 18 hours. Cell viability was measured using the MTT assay (see Materials and Methods) and expressed as OD at 575 nm. *Panel B: *Macrophages were incubated as described above. NO production was measured as nitrite concentration in the culture media as described in Materials and Methods. Mean values not sharing a common letter are significantly different (p < 0.05).

Polymyxin B is an antibiotic drug, which selectively binds and neutralizes LPS. Since LPS enhances CEES toxicity, we tested the ability of polymyxin B to reduce CEES toxicity (500 μM) and decrease NO generation in LPS (50 ng/ml) stimulated macrophages. Figure 7a shows that polymyxin B (10 μg/ml) had no cytotoxic effect on RAW 264.7 macrophages but partially reduced the cytotoxicity of CEES+LPS treated cells (18 h). Nevertheless, polymyxin B produced at least a six fold increase in cell viability compared to cell treated with both LPS and CEES for 18 h. As shown in Figure 7b, polymyxin B effectively blocked the production of NO (measured as nitrite levels) in LPS (50 ng/ml for 18 h) treated macrophages as would be expected if it bound and blocked the action of LPS.

## Discussion

The cytotoxic effect of HD, and its analogue CEES, is believed to involve an increased generation of damaging free radicals and ROS [8,11-13,32]. The data presented here show that LPS in combination with CEES induces intracellular GSH and thiol depletion as well as increased levels of protein carbonyls. In experiments with various human cell lines we have found that GSH depletion is relatively rapid as it occurs within first hour of incubation (data not shown). Thus, it is likely that this depletion is due, in large part, by a direct reaction of CEES with GSH. The measurement of protein carbonyls is one of the best indices for oxidative stress due to the stability of protein carbonyls and sensitivity of the measurement [33]. Cellular thiols are important markers of the redox state of the cell. In particular, GSH is one of the major components of the intracellular redox system and a key intracellular antioxidant that functions as a substrate for glutathione peroxidase which detoxifies both hydrogen peroxide and lipid hydroperoxides [34,35]. Depletion of intracellular stores of GSH plays an important role in the development of oxidative stress [12,13,36]. Recent work also suggests that the anti-apoptotic protein Bcl-2 directly interacts with GSH to regulate an important mitochondrial GSH pool that influences mitochondrial oxidative stress and subsequent apoptosis [37]. It is highly possible that both sulphur and nitrogen mustards possess a similar ability to deplete cellular thiols and induce protein oxidation.

Taken together, our data strongly suggest that CEES induces oxidative stress in stimulated macrophages. Moreover, the pattern of oxidative stress parallels the pattern observed for CEES cytotoxicity, i.e., cytotoxicity and oxidative stress are amplified in cells treated with both CEES and LPS. The addition of 5–10 mM NAC, a well characterized water-soluble antioxidant, was found to be very effective in minimizing CEES toxicity in stimulated macrophages and in preventing GSH depletion. Our data suggests that NAC can be added five hours before or even five hours after CEES and still exert a cytoprotective effect. Das et al. [9] recently found that NAC in drinking water was effective in reducing CEES-induced lung toxicity to Guinea pigs. Fan et al. [38] have shown that liposomal encapsulated NAC delivered intratracheally was more effective than free NAC against acute respiratory distress syndrome in a rat model. It is interesting, therefore, that McClintock et al. [39] have shown that reducing agents (NAC or GSH), as well as some anti-oxidant enzymes, delivered via liposomes, can substantially diminish CEES-induced injury in rat lungs. We are currently formulating an optimal antioxidant liposome preparation for treating either lung or skin induced CEES/HD injury.

We previously reported that CEES induces a transient loss of iNOS protein expression in LPS stimulated RAW 264.7 macrophages but does not inhibit the enzymatic activity of iNOS. Based of the work of others [22-25], we hypothesized that NAC treatment would not only be protective against CEES toxicity but would also restore NO production in LPS stimulated macrophages treated with CEES. Our results indicate, however, that this was not the case. Our data did, however, show that NAC effectively increases cell viability, increases GSH levels and reduces oxidative stress in LPS stimulated macrophages treated with CEES.

CEES could inhibit iNOS protein synthesis by a number of possible molecular mechanisms which we are currently exploring [18]. It is generally accepted that both the transcription factor NF-κB and STAT-1 play central roles in the LPS induction of iNOS [17,40]. It is possible that CEES/HD could inhibit the NF-κB and/or the STAT-1 pathways in RAW 264.7 macrophages and consequently block iNOS gene expression. For instance, CEES could alkylate the NF-κB consensus nucleotide binding sequences thereby preventing the binding of activated NF-κB to the iNOS promoter and block the subsequent production of iNOS mRNA and protein expression. Previous studies *in vitro *have shown that DNA alkylation by CEES [41,42] or by nitrogen mustard [43] can inhibit the DNA binding of transcription factor AP2 or NF-κB.

Alternatively, the DNA binding ability of the NF-κB and/or STAT-1 transcription factors could be reduced by direct covalent modification by CEES or as an indirect result of GSH depletion, i.e., redox regulation. Nishi et al. [44] have found, for example, that the cysteine-62 (Cys-62) residue of the p50 NF-κB protein subunit is oxidized in the cytoplasm but reduced in the nucleus, and that the reduced form is essential for NF-κB DNA binding. It is possible that CEES could rapidly react with Cys-62 of the p50 NF-κB subunit and prevent its DNA binding. However, since NAC was found to restore GSH levels without restoring iNOS activity (see Figures 1 and 7), it is unlikely that a GSH redox modulation of the p50 Cys-62 is the molecular mechanism for CEES induced loss of iNOS protein in LPS-stimulated macrophages. This cannot, however, be completely ruled out based on our current data.

Moreover, there is evidence suggesting that alkylating agents do not inhibit but rather promote NF-κB activation. It is known that CEES or HD treated cells release elevated levels of TNF-α and also show NF-κB activation both *in vitro *and *in vivo *as measured by electrophoretic mobility shift assays (EMSAs)[7,45,46]. Minsavage and Dillman recently demonstrated that NF-κB is activated by HD treatment in human cell lines via nonclassical p53-dependent pathway [47]. Collectively, these data suggest that the inhibition of iNOS expression by CEES or HD could be due to downregulation of the STAT-1 and/or classical NF-κB pathway. We are currently exploring these various molecular mechanisms.

In the work presented here, we also tested the ability of polymyxin B to block the effect of LPS. Polymyxin B binds to the lipid A domain of LPS and neutralizes its activity. Our data show that polymyxin B effectively inhibits CEES toxicity in LPS stimulated cells. *In vivo*, LPS could directly enhance CEES/HD toxicity in cells with functional CD14 receptors or by triggering the release of pro-inflammatory cytokines, such as TNF-α and IL-1β, by immune cells. We have previously demonstrated that inflammatory cytokines also enhance CEES cytotoxicity [15].

## Conclusion

Our *in vitro *work presents novel evidence supporting the view that oxidative stress is an important component of CEES/HD toxicity and that antioxidants have therapeutic potential. We anticipated that NAC would prevent GSH depletion and restore the loss of iNOS activity in CEES treated macrophages stimulated with LPS. Although NAC was effective in preventing both CEES toxicity and GSH depletion, it failed to restore iNOS expression. Our results to date indicate that CEES causes a transient decrease in iNOS protein syntheses rather than a direct inhibition of iNOS activity due to covalent modification(s) by CEES. We are currently investigating the molecular mechanism(s) for the down regulation of iNOS expression by CEES.

Inhibition of iNOS and NO production could be an important element in the slow wound healing observed subsequent to CEES/HD injury. Considerable evidence suggests that iNOS is an important component of wound healing [19,20,48]. Although NAC maybe effective at reducing CEES/HD toxicity it is not effective at elevating NO production due to iNOS inhibition by CEES/HD. A more detailed understanding of the molecular mechanism(s) responsible for iNOS inhibition by CEES/HD could, therefore, be useful in the design of more effective countermeasures.

The fact that LPS was found to enhance CEES toxicity highlights the potential importance of preventing secondary infection in the treatment of HD toxicity.

LPS is a component of gram negative bacteria and a ubiquitous environmental contaminant. Its presence at very low levels (ng/ml) amplifies the toxicity of CEES. Polymyxin B, a topically applied antibiotic that binds LPS, was shown to block the iNOS inducing ability of LPS and to reduce CEES toxicity in LPS stimulated cells. Polymyxin B could, therefore, be useful as a supportive treatment in order to prevent secondary infections and to reduce HD toxicity, since it both neutralizes LPS and prevents the growth of gram-negative bacteria in healing wounds.

The path to an optimal countermeasure to CEES/HD exposure may lie in a poly-drug formulation that minimizes oxidative stress, prevents inflammation and secondary infections, and, also, protects iNOS activity. Antioxidant liposomes are currently being investigated as they have unique ability for targeted delivery of both water-soluble and lipid soluble antioxidants [49] or other drugs, for instance, polymyxin B (personal communications, Dr. Zach Suntres) as well as anti-inflammatory agents.

## Methods

### Materials

RPMI-1640 medium without phenol red and fetal bovine serum with a low endotoxin level were purchased from Life Technologies (Gaithersburg, MD). *Escherichia coli *lipopolysaccharide serotype 0111:B4, 3-(4,5-dimethylthiazolyl-2)-2,5-diphenyltetrazolium bromide (MTT), CEES, NAC, Greiss reagent, GSH, BHT, EDTA, and all organic solvents used were obtained from Sigma Chemical Company (St. Louis, MO). Fluorescent dyes carDCFH-DA, CMAC, and CMF-DA were purchased from Molecular Probes (Invitrogen Corp., Carlsbad, CA).

### Cell culture and treatments

RAW264.7 murine macrophage-like cells (American Type Culture Collection, Rockville, MD) were cultured at 37°C in a humidified incubator with 5% CO_2 _in RPMI-1640 medium with 10% fetal bovine serum, 100 U/ml penicillin and 100 mg/ml streptomycin (GiBcoBRL Grand Island, NY). Adherent cells were subcultured in 96 well Costar tissue culture plates and treated with CEES and/or LPS in the presence or absence of various concentrations of NAC as indicated in the Figure legends. CEES was used only as a fresh 50 mM stock solution in anhydrous ethanol. LPS was prepared as a 0.5 μg/ml stock solution in PBS, filter-sterilized and stored at -20°C for up to 6 months. NAC was prepared as a 0.5 M stock solution in PBS (pH adjusted to 7.4), filter-sterilized and stored at 4°C for up to two weeks.

### MTT assay

MTT assay was performed by a slight modification of the method described by Wasserman et al. [50,51]. Briefly, at the end of each experiment, cultured cells in 96 well plates (with 200 μl of medium per well) were incubated with MTT (20 μl of 5 μg/ml per well) at 37°C for 4 h. The formazan product was solubilized by addition of 100 μl of dimethyl sulfoxide (DMSO) and the OD measured at 575 nm with a Spectramax Plus 384 microplate reader (Molecular Devices Corp, Sunnyvale, CA)

### NO generation in RAW264.7 macrophages

The production of NO, reflecting cellular NO synthase activity, was estimated from the accumulation of nitrite (NO_2_^-^), a stable breakdown product of NO, in the medium. NO_2_^- ^was assayed by the method of Green et al. [52]. Briefly, an aliquot of cell culture medium was mixed with an equal volume of Greiss reagent which reacts with NO_2_^- ^to form an azo-product. Absorbance of the reaction product was determined at 532 nm using a Spectramax Plus 384 microplate reader (Molecular Devices Corp, Sunnyvale, CA). Sodium nitrite was used as a standard to calculate NO_2_^- ^concentrations.

### Quantitative GSH analyses

RAW264.7 macrophages incubated in 96-well plate (~10^6 ^adherent cells/well) and treated with LPS/CEES/NAC as indicated in the Figure legends was assayed for total GSH (GSH plus GSSG) using the GSH assay kit (World Precision Instruments, Sarasota, FL) according to the company's protocol. This assay uses the Tietze's enzymatic recycling method [53]. In order to measure just GSSG, 2-vinylpyridine was first used to derivatize GSH alone [54]. Total GSH and GSSG levels were normalized to the total protein (as determined by the standard BCA assay). Alternatively, GSH analyses of the cell lysates were analyzed by isocratic HPLC with electrochemical detector composed of Coulochem II model 5200A and a Coulochem 5011 analytical cell (ESA Inc, Chelmsford, MA) as described by [55]. Since the cell lysates contained no measurable levels of homocysteine, this aminothiol was used as an internal standard.

### Protein carbonyl measurement

Protein carbonyl levels were measured by an enzyme immunoassay kit from Cell Biolabs (San Diego, CA) according to the manufacture's instructions. In this assay, the protein samples are derivatized by making use of the reaction between 2,4-dinitrophenylhydrazine (DNPH) and protein carbonyls to form a DNP hydrazone which is assayed using an anti-DNP antibody and a HRP conjugated secondary antibody. A standard curve from the oxidized BSA standards was run with each microplate. This kit assay is essentially a modification [56] of the method described by Buss et al. [57].

### Fluorescent microscopic analyses

The cell density was adjusted to 2 × 10^5^/ml, and a 100 μl aliquot of the cell suspension in media was placed in each well of an 8-well Lab-Tek chamber glass slide (Nunc, Rochester, NY). Vehicle alone, CEES alone (500 μM), CEES+LPS (50 ng/ml) in the presence or absence of NAC (10 mM) were simultaneously added to chamber slides and incubated for 12 h at 37°C in 5% CO_2_. At the end of the treatment a stock solution of desired fluorescent probe in DMSO was added and the slides incubated for an additional 30 min at 37°C. The cells were washed with fresh PBS twice, observed and digitally photographed using a MOTIC inverted phase contrast fluorescence microscope equipped with a Nikon Coolpix E4300 4-megapixel camera (Martin Microscope, Easley, SC). A 20 μM carDCFH-DA and a standard FITC filter were used to monitor combined ROS and RNOS generation; a 20 μM CMAC and a standard DAPI filter were used to monitor intracellular GSH; a 20 μM CMF-DA and a standard FITC filter were used to monitor cellular thiol levels. All the optical filters were obtained from Chroma Technology Corp (Rockingham, VT).

### Statistical analyses

Data were analyzed ANOVA followed with the Scheffe test for significance with p < 0.05 using SPSS 14.0 for Windows (Chicago, IL). Results were expressed as the mean ± SD. In all the Figures, mean values not sharing a common letter are significantly different (p < 0.05). Mean values sharing a common letter are not significantly different. The mean values and standard deviations of at least three independent experiments are provided in all the Figures.

## Abbreviations

AP2, activating protein 2; CEES, 2-chloroethyl ethyl sulphide; carDCFH-DA, carboxy-dichlorofluorescin diacetate; CMAC, 7-amino-4-chloromethylcoumarin; CMF-DA, 5-chloromethylfluorescein diacetate; DNPH, 2,4-dinitrophenylhydrazine; GSH, reduced glutathione; GSSG, oxidized glutathione; HD, sulphur mustard gas; IL-1β, interleukin-1 beta; LPS, lipopolysaccharide; MTT, 3-(4,5-dimethylthiazool-2yl)-2,5-diphenyltetrazolium bromide; NAC, N-acetyl-L-cysteine; NO, nitric oxide; iNOS, inducible nitric oxide synthase; NF-κB, nuclear factor kappa B; RNOS, reactive nitrogen oxide species; ROS, reactive oxygen species; STAT-1, signal transducer and activator of transcription-1; TNF-α, tumor necrosis factor-alpha

## Authors' contributions

VP and WLS analyzed the data and drafted the manuscript. WLS supervised the overall conduct of the research, which was performed in his laboratory. VP, MQ and HY carried out the experimental work in this study and performed the statistical analyses. MS (along with WLS) conceived of the study, participated in the study design, and provided continuous evaluation of the experimental data. All authors read and approved the final manuscript.
